# mRNA-based COVID-19 vaccines appear not to increase immune events in cancer patients receiving immune checkpoint inhibitors

**DOI:** 10.2217/fvl-2021-0166

**Published:** 2021-09-02

**Authors:** Emre Yekedüz, Rüveyda Ayasun, Elif Berna Köksoy, Güngör Utkan, Yüksel Ürün, Hakan Akbulut

**Affiliations:** 1 ^1^Department of Medical Oncology, Ankara University, Ankara, Turkey; 2 ^2^Ankara University Cancer Research Institute, Ankara, Turkey; 3 ^3^Hacettepe University Cancer Institute, Department of Medical Oncology, Ankara, Turkey

**Keywords:** cancer, COVID-19, immunotherapy, mRNA, SARS-CoV-2, vaccine

We are in the pandemic’s second year, and vaccines against COVID-19 have started rolling out in early December 2020. To date, two mRNA-based vaccines were granted for an emergency use authorization by the US FDA [1]. It is a relatively new vaccine development technology and these vaccines are considered to have some advantages, such as rapid development process, lower costs and more potent effects (e.g., increased immunogenicity), compared with conventional inactive vaccines [2]. Nucleic acid vaccines, including plasmid DNA or mRNA, induce pro-inflammatory responses. The pro-inflammatory potential of mRNA, which is more prominent than DNA, provides a self-adjuvant activity and makes mRNA a popular vaccine candidate against viral infections. However, vigorous pro-inflammatory activity of mRNA *per se* results in both local and systemic inflammation, including autoimmune responses, particularly when administered in lipid nanoparticles [3].

mRNA-based vaccines are not solely designed against viral infections. There have been ongoing studies to develop mRNA-based vaccines against certain types of cancer, in particular over the past 5 years. Up to now, there was no mRNA-based vaccine approved by the FDA, in turn, we do not have any safety information about the utilization of these vaccines in cancer patients. However, being inactivated is one of the main advantages of mRNA vaccines in immunocompromised patients. We have sufficient experience for the use of inactivated vaccines in cancer patients treated with chemotherapeutics. Fortunately, there were no safety concerns regarding the use of conventional inactivated vaccines in these patients [4]. In parallel, recent studies demonstrated no increase in incidence or severity of immune-related adverse events (irAEs) for the vaccination of cancer patients receiving immune checkpoint inhibitors (ICIs) with inactivated vaccines [5,6].

Autoimmunity is considered as a safety concern of mRNA-based vaccines due to the Toll-like receptor 7-mediated activation of type I interferon response [2]. Not surprisingly, a growing body of literature has raised the hypothesis of an increased risk of autoimmune diseases following COVID-19, particularly in susceptible patients [7]. In the Phase III trial of the BNT162b2 mRNA COVID-19 vaccine, 3.9% of all patients in the vaccine arm had a malignancy. However, there was no information as to whether patients treated with immunotherapy were included in this trial [8]. Moderna’s product named mRNA-1273 COVID-19 vaccine has shown to be protective against severe COVID-19 infection and to reduce hospitalization, in turn, was recently approved for emergency use by the FDA [1]. Similar to Pfizer’s vaccine, we do not have detailed information about the characteristics of the participants and whether they received any ICIs during or prior to the clinical trial. Immunotherapy and ICIs play an important role in the battle against cancer. Following the emergency use authorization of COVID-19 vaccines, cancer patients will be vaccinated promptly due to the higher mortality rates of COVID-19 in this patient population [9,10]. In this case, a safety concern is arising as to whether the risk for irAEs in cancer patients receiving ICIs and vaccinated with an mRNA-based COVID-19 vaccine is increased. To date, this notion is yet to be determined. Therefore, vaccination of cancer patients under ICI therapy should be rigorously interrogated by healthcare providers and policy makers prior to starting of mass vaccination. In a study including malign melanoma patients, a combination of mRNA-based vaccine against melanoma named FixVac and anti-programmed death 1 (anti-PD-1) inhibitor demonstrated an effective response without any serious irAEs. The main adverse events in this study were flu-like symptoms which were resolved within 24 h [11]. Close monitoring of cancer patients treated with ICIs during and prior to the vaccination is the easiest way to get information about whether there is an increased risk for irAEs in those patients. For instance, pneumonitis is one of the most common adverse events observed in patients under ICI therapy. Given the profound activation of innate and adaptive immunity upon mRNA vaccines, there might be a possibility of antibody dependent enhancement which causes pulmonary damage. Therefore, medical oncologists should be well prepared for any unintended side effect in patients that have previously developed irAEs following to ICI therapy. Besides, scheduling of vaccination of this patient population should be optimized by prospective studies. Data from cancer patients receiving ICIs and vaccinated with Pfizer BNT162b2 mRNA vaccine has been published recently [12]. According to this data, no increased risk of irAEs was observed after vaccination in cancer patients treated with ICIs. However, this result came from a relatively short-term observational study. Of note, muscle pain was more commonly observed in patients treated with ICIs than the healthy control group. Except muscle pain, no additional safety concern was recorded in patients treated with ICIs [12]. Indeed, these findings may encourage physicians and scientists to perform mRNA-based COVID-19 vaccines without concerns. Nevertheless, we need long-term data from the large patient cohorts.

On the other hand, these two vaccines encode different viral proteins in the antigen-presenting cells. Pfizer’s vaccine encodes full-length spike proteins consisting of both S1 including receptor binding domain and S2 domains. In contrast, Moderna’s vaccine encodes solely S2 domain of spike protein which mediates membrane fusion but not receptor binding of the virus [8,13]. Given the functional and structural differences, neutralizing antibody titers in rabbits immunized with S2 subunit were much lower than rabbits immunized with either S1 subunit or receptor binding domain. Yet another explanation of this difference is the limited accessible site of S2 domain due to substantial N-glycan shielding [14]. At this point, a new question arises as to whether this difference may affect the severity of irAEs of ICIs in cancer patients. We are awaiting the results regarding the severity of irAEs of cancer patients receiving ICIs from the vaccine arm of Phase III trials, if applicable. Beyond that we should be vigilant when vaccinating cancer patients receiving ICIs and should collect the data regarding the aforementioned adverse effects.

## Future perspective

In the second year of the pandemic, the rates of vaccination with the COVID-19 vaccines are increasing at an incredible pace across the world. Cancer patients take place in the susceptible group to the COVID-19. In this regard, they have a priority for COVID-19 vaccination in many countries. However, they are treated with different anticancer therapies such as chemotherapy, radiotherapy, targeted therapies and ICIs. We do not have enough data regarding the long-term effects of mRNA-based COVID-19 vaccines in cancer patients treated with ICIs. In the near future, we hope to see the results of real-world studies regarding the long-term effect of mRNA-based COVID-19 vaccines on cancer patients treated with ICIs. One of the most common concerns about using mRNA-based COVID-19 vaccines in patients treated with ICIs is irAEs. In parallel to the increased vaccination rates of cancer patients, the real-world data will show the long-term safety of mRNA-based COVID-19 vaccines in cancer patients treated with ICIs.
